# Oleic Acid Inhibits SDC4 and Promotes Ferroptosis in Lung Cancer Through GPX4/ACSL4

**DOI:** 10.1111/crj.70014

**Published:** 2024-10-13

**Authors:** Jingfei Dong, Fei Qi, Huiqing Qie, Shibu Du, Li Li, Yan Zhang, Kaiyue Xu, Dehui Li, Yapei Xu

**Affiliations:** ^1^ Department of Clinical Laboratory Hebei Provincial Hospital of Chinese Medicine Shijiazhuang Hebei China; ^2^ School of Basic Medical Sciences Chengde Medical University Chengde Hebei China; ^3^ Department of Health Care Hebei General Hospital Shijiazhuang Hebei China; ^4^ Department of Functional Medicine Hebei Provincial Hospital of Chinese Medicine Shijiazhuang Hebei China; ^5^ Department of Oncology Hebei Provincial Hospital of Chinese Medicine Shijiazhuang Hebei China; ^6^ Gastrointestinal Endoscopy Room Hebei Provincial Hospital of Chinese Medicine Shijiazhuang Hebei China

**Keywords:** ACSL4, ferroptosis, GPX4, lung cancer, oleic acid, SDC4

## Abstract

**Introduction:**

As a common malignancy, lung cancer has a relatively poor prognosis and a low survival rate. In recent years, ferroptosis, as an emerging filed, has great promise in the potential treatment of cancer. *Brucea javanica* oil (BJO) is often used to treat various cancers. Oleic acid (OA) is the main ingredient of BJO. In this study, we investigated the role and molecular mechanism of OA in lung cancer treatment by promoting ferroptosis.

**Methods:**

In this study, A549 cells and H1299 cells were used for in vitro experiments, and a CCK‐8 test, scratch test, and MTT experiment were carried out. We examined reactive oxygen species (ROS), the JC‐1 probe, glutathione (GSH) expression, lipid peroxidation, SDC4 mRNA levels, and ACSL4, SLC7A11, GPX4, and SDC4 protein levels.

**Results:**

The results showed that OA could inhibit the proliferation and migration of A549 cells and H1299 cells, SDC4 was a potential therapeutic target of OA against lung cancer, and OA treatment significantly inhibited the expression of SDC4 in A549 cells and H1299 cells. OA induces ferroptosis in A549 cells and H1299 cells, decreases GSH levels, increases lipid peroxidation levels, and decreases SDC4 mRNA expression; in addition, OA upregulates ACSL4 expression and decreases SLC7A11, GPX4, and SDC4 expression.

**Conclusion:**

This study confirmed that OA could inhibit SDC4 expression and promote the occurrence of ferroptosis in A549 cells and H1299 cells through the GPX4/ACSL4 pathway, providing an effective basis for the use of drugs targeting ferroptosis in lung cancer treatment.

## Graphical abstract

In this study, in vitro experiments on A549 cells and H1299 cells confirmed that oleic acid can inhibit the expression of SDC4, promote the occurrence of ferroptosis in A549 cells and H1299 cells through the GPX4/ACSL4 pathway, and provide an effective basis for the clinical application of targeted ferroptosis drugs in the effective treatment of lung cancer.

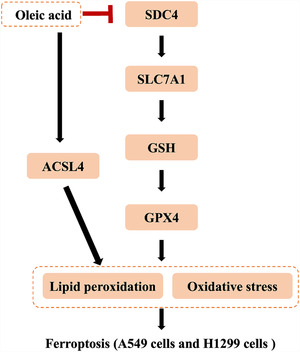

## 1 Introduction

As a common malignant tumor, lung cancer has a high incidence and mortality rate and is an important factor threatening human health [1, 2, 3]. There are two main categories of lung cancer, namely small cell lung cancer (SCLC) and non‐SCLC (NSCLC), with NSCLC being the most common type. The main available treatments include surgery, chemotherapy, and radiotherapy. Despite advancements in treatment modalities, lung cancer is highly metastatic and prone to drug resistance and radiation tolerance, resulting in unsatisfactory overall survival. Therefore, alternative therapeutic options for lung cancer need to be explored.

Induction of cell death is an emerging method for cancer treatment. Programmed cell death (PCD) includes apoptosis, pyroptosis, autophagy, and other forms and is involved in the development and homeostasis of organisms [2]. Ferroptosis, caused by iron overload, is also one of them, and like other PCD patterns, it is regulated by genetics and multiple signaling pathways [4]. Iron is an indispensable trace element in life activities, but excessive iron ions can trigger cell death by inducing oxidative stress reactions in cells. Studies have shown that ferroptosis can be involved in the development of various diseases, especially tumors and neurodegenerative diseases [3]. The mechanism of ferroptosis includes two main processes: excessive accumulation of iron ions and increased intracellular oxidative stress. Excessive accumulation of iron ions can occur through multiple pathways, such as increased uptake of iron ions, increased endogenous release of iron ions, or excessive supply of exogenous iron ions [5]. The increase in intracellular oxidative stress is due to the participation of iron ions in the generation of free radical reactions, leading to increased intracellular oxidative damage [6]. These processes work together to cause cellular dysfunction and cell death. In tumors, ferroptosis is also considered an important therapeutic strategy [7]. By increasing the accumulation of intracellular iron ions, ferroptosis can be induced in tumor cells, thereby achieving the effect of treating tumors.

The SDC4 gene encodes the classical epithelial cell adhesion molecule 4 (syndecan4), which is involved in various pathophysiological processes. SDC4 belongs to the protein family with the glycosaminoglycan side chain in the extracellular matrix, and it is also a kind of cell membrane glycoprotein, which acts as a bridge between cells and the extracellular matrix [8, 9]. It is involved in cell migration and adhesion during embryonic development. In addition, SDC4 is also involved in extracellular matrix remodeling, tumor cell metastasis and invasion, angiogenesis, cardiovascular disease, inflammatory response, and muscle regeneration. Many diseases are strongly associated with the aberrant expression of the SDC4 gene [10, 11]. SDC4 is involved in tumor growth as the lung cancer tumor size decreases in SDC4 KO mice [12]. SDC4 expression was increased after lung injury and tumor cell seeding [13, 14]. Silencing the SDC4 gene in human papillary thyroid cancer can inhibit epithelial mesenchymal transition and promote apoptosis through the Wnt/β‐catenin signaling pathway in humans [15]. However, the relationship between ferroptosis and SDC4 has been poorly studied and is not yet clear. Therefore, whether SDC4 can participate in lung cancer treatment by inducing ferroptosis urgently needs a more precise analysis.

Traditional Chinese medicine (TCM) has been shown to be effective in cancer treatment and can prolong survival by improving immune function [16]. Oleic acid (OA) is the main active ingredient of *Brucea javanica* oil (BJO), which is extracted from seeds of the herb *Brucea javanica* [17, 18]. BJO or OA can be used to treat a variety of cancers such as prostate cancer, breast cancer, and colorectal cancer, and OA can also be combined with chemotherapy [16, 19]. However, little is known about its targets and action mechanism in lung cancer.

To investigate the anticancer effect and mechanism of OA on lung cancer, we first stimulated lung cancer cells with OA in vitro and observed its changes and further analyzed the role of ferroptosis, the SDC4 gene, and the glutathione peroxidase 4 (GPX4)/ACSL4 signaling pathway. The results showed that OA had a significant anticancer effect on lung cancer cells. It can promote ferroptosis by regulating the SDC4 gene and GPX4/ACSL4 pathway.

## 2 Materials and Methods

### 2.1 Cell Culture

Lung cancer cell lines A549 and H1299 were purchased from Procell Life Science & Technology Co., Ltd. The cells were grown in Ham's F‐12K Medium (Gibco, Thermo Fisher Scientific, Inc.) containing 10% FBS (Gibco, Thermo Fisher Scientific, Inc.) and 1% antibiotics (penicillin and streptomycin) in an incubator at 37°C with 5% CO_2_.

### 2.2 Cell Counting Kit‐8 (CCK‐8)

A549 cells and H1299 cells treated with OA (Cat. No. O815203, MACKLIN) were seeded into a 96‐well plate, with 3 × 10^3^ cells per well and cultured for 24 h. At the indicated time points, 10 μL of CCK‐8 (Cat. No. C0043, Beyotime) was added and incubated for an additional 2 h. Absorbance values were determined at a wavelength of 450 nm using a microplate reader (Flash, China).

### 2.3 Wound‐Healing Assay

OA‐treated cells (2 × 10^5^ total cells per well) were seeded on 24‐well plates and incubated for 24 h. When the cells are 90% confluent, they were scratched with a 200‐μL sterile tip. The debris was gently washed, the width of each scratch was recorded, and the time point was set as 0 h. Next, fresh Ham's F‐12K without serum was added to it and incubated for an additional 24 h. Subsequently, the width of the scratch at 12 and 24 h was recorded. The relative mobility was detected using an inverted light microscope (SOPTOP ICX4I, China) at a magnification of × 100.

### 2.4 Western Blot

The cells were lysed in RIPA Lysis Buffer (Cat. No. R0010, Solarbio). Approximately 30 μg of total protein extracts was separated using 10% SDS‐PAGE and then transferred to a polyvinylidene difluoride (PVDF) membranes (Cat. No. 03010040001, Roche). After the membrane was blocked with 5% skim milk for 1 h, it was incubated overnight with primary antibodies against SDC4 (Cat. No. AF0831; dilution, 1:1000; Affinity), GPX4 (Cat. No. DF6701; dilution, 1:1000; Affinity), ACSL4 (Cat. No. DF12141; dilution, 1:1000; Affinity), SLC7A11 (Cat. No. DF12509; dilution, 1:500; Affinity), β‐actin (Cat. No. WL01372; dilution, 1:500; Wanleibio) at 4°C. The horseradish peroxidase–linked secondary antibodies were incubated for 1 h and were detected using an electrochemical luminescence (ECL) reagent. Images were acquired using the chemiluminescence imaging system JP‐K600 (Jiapeng, China).

### 2.5 RNA Isolation and Real‐Time PCR

Total RNA was isolated using a TRIzol reagent (Cat. No. 9108, TaKaRa), and the RNA concentration was detected using an ultraviolet spectrophotometer (Flash, China). Next, 1 μg of RNA was reverse transcribed (50°C for 15 min, 85°C for 5 s) using the HiScript III all‐in‐one RT SuperMix (Cat. No. R333‐01, Vazyme). Real‐time PCR was performed using HiScript II One‐Step qRT‐PCR SYBR Green Kit (Cat. No. Q221‐01, Vazyme) and X 960 Real‐time PCR (Heal Force, China) in a total volume of 20 μL, at 95°C for 30 s, 40 cycles of 95°C for 5 s and 60°C for 20 s. The primer sequences are shown in Table 1. The relative mRNA levels were calculated by the 2^−△△Cq^ method.

**TABLE 1:** The qRT‐PCR primer sequences are listed.

	Sequence (5′ → 3′)	
SDC4	Forward prime	GUAUCUCCAGCUCUGAUUATT
	Reverse prime	UAAUCAGAGCUGGAGAUACTT
β‐Actin	Forward prime	GTGCTATCCCTGTACGCCTC
	Reverse prime	AGGTAGTCAGTCAGGTCCCG

### 2.6 Detection of Reactive Oxygen Species (ROS)

DCFH‐DA was diluted with serum‐free medium at 1RV 1000 to a final concentration of 10 μmol/L, and an appropriate amount was added to the cell culture medium. It is appropriate to add the volume to fully cover the cells. The solution was incubated at 37°C for 20 min and washed three times to completely remove excess DCFH‐DA.

OA‐ or si‐SDC4–treated cells were seeded onto 24‐well plates totaling 2 × 10^5^ per well and incubated for 24 h. DCFH‐DA (Cat. No. S0033S, Beyotime) was added to the working solution and then incubated at 37°C for 30 min. A laser scanning confocal microscope (Leica, China) used a 488‐nm excitation wavelength and 525‐nm emission wavelength to measure fluorescence intensity.

### 2.7 Mitochondrial Transmembrane Potential Measurement

OA‐ or si‐SDC4–treated cells were seeded onto 24‐well plates and incubated for 24 h. The levels of mitochondrial transmembrane potential (MMP) were detected by JC‐1 fluorescence staining (Cat. No. C2003S, Beyotime). A JC‐1 dyeing solution of 1 mL was added and mixed thoroughly. Cells were incubated at 37°C for 20 min. The supernatant was aspirated and washed twice with buffer; then, 2 mL of the cell culture medium was added. JC‐1 in healthy mitochondria forms red‐fluorescent aggregates whereas monomers form green‐fluorescent aggregates in depolarized mitochondria. Under a laser scanning confocal microscope, an excitation light wavelength of 490 nm and an emission light wavelength of 530 nm are used to detect the fluorescence intensity (Leica, China). The results are expressed as a percentage of the intensity of green fluorescence.

### 2.8 Detection of Malondialdehyde (MDA)

Cells with different treatments were collected into a centrifuge tube, and the supernatant was discarded after centrifugation. An extractive solution of 1 mL was added to every five million cells, and the cells were crushed by ultrasonic wave (power 200 W, ultrasonic wave for 3 s, interval 10 s, repeated 30 times) and centrifuged at 8000 *g* at 4°C for 10 min; the supernatant was taken and put on ice for testing. The microplate reader was prewarmed for more than 30 min and adjusted to zero with distilled water. A mixture of 300 μL of MDA detection working solution, 100 μL of sample, and 100 μL of reagent III was subjected to a water bath at 100°C for 60 min and ice bath cooling and was centrifuged at 10 000 *g* for 10 min at room temperature. In a 96‐well plate, 200 μL of supernatant was absorbed, and its absorbance was determined at 532 and 600 nm.

### 2.9 Detection of Reduced Glutathione (GSH)

There were 1 × 10^6^ cells of different treatments collected into centrifuge tubes, and the supernatant was discarded after centrifugation. The cells were crushed by sonication in an ice bath (power 200 W, sonication for 3 s, 10‐s interval, repeated 30 times) and centrifuged at 8000 *g* for 10 min, and the supernatant was placed on ice for detection. The microplate reader was prewarmed for more than 30 min and adjusted to zero with distilled water. A standard solution of 10 mg/mL was absorbed and diluted with distilled water to 300, 200, 100, 50, and 25 μg/mL to prepare standards with different concentrations. We mixed 20 μL of the sample and standard with 40 μL of reagent II and 140 μL of reagent III, respectively, and let it stand for 2 min at room temperature. The absorbance rates of the measuring tube, the standard tube, and the blank tube at 412 nm were determined.

### 2.10 Statistical Analysis

All data are presented as mean ± standard deviation (*SD*). The GraphPad Prism 9.0 (GraphPad software Version 9.0.1, LLC, CA, United States) was used for statistical analysis. When two groups were compared, one‐way analysis of variance (ANOVA) followed by the least significant difference (LSD) test was used. *p* values < 0.05 were considered statistically significant.

## 3 Results

### 3.1 OA Inhibits the Proliferation and Migration of A549 Cells and H1299 Cells

**FIGURE 1:**
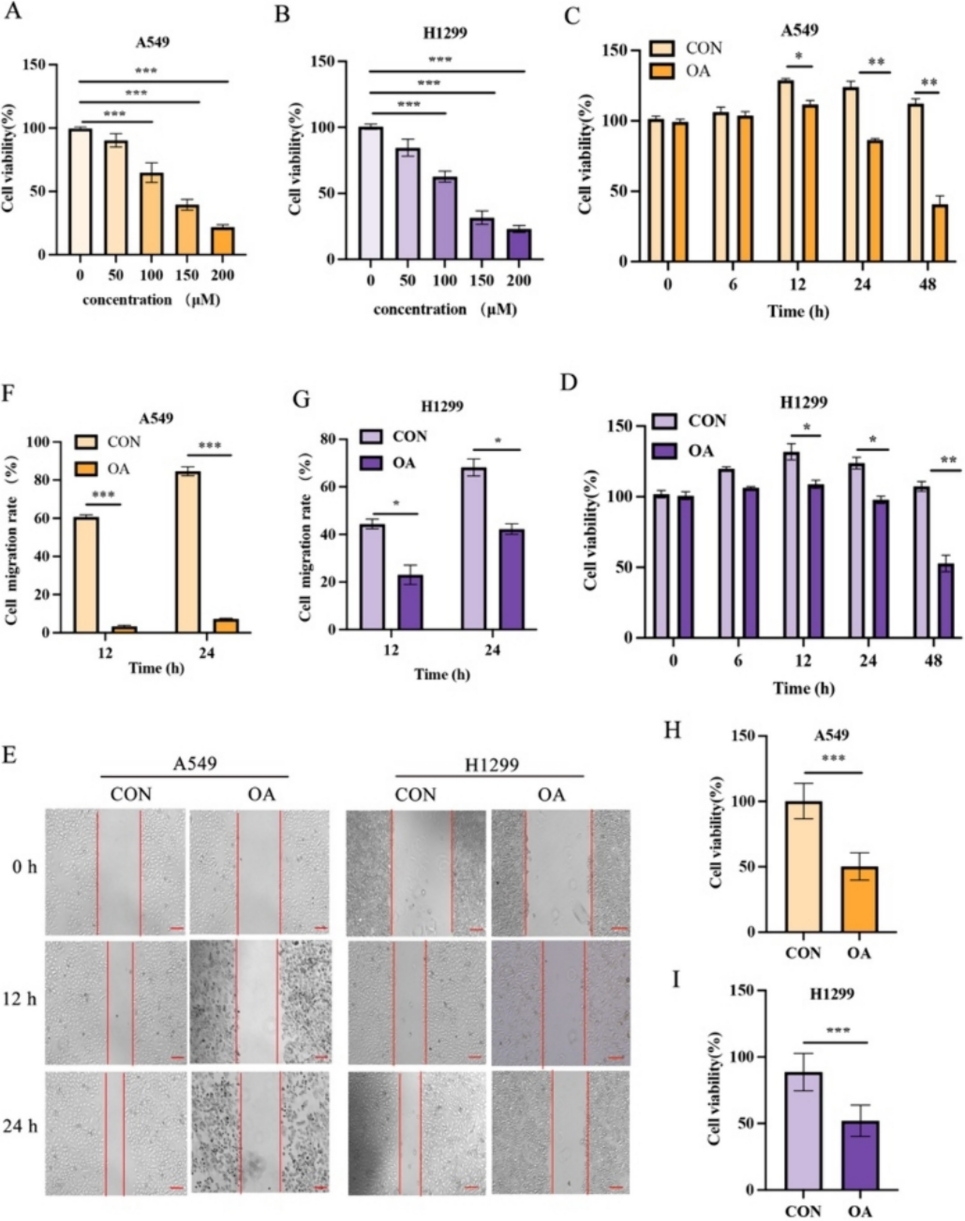
OA inhibits cell proliferation and migration. (A, B) Effects of different concentrations of OA on the viability of A549 cells and H1299 cells detected by CCK‐8. (C, D) Effect of 100 μM of OA on the viability of A549 cells and H1299 cells at different times detected by CCK‐8. (E) Observation of wound healing under a microscope. (F, G) Graphical data representing the percentage of migrated area determined by the wound‐healing test. (H, I) Effect of 100 μM of OA on the viability of A549 cells and H1299 cells at 12 h detected by MTT. *n* = 3. *p < 0.05, **p < 0.01, and ***p < 0.005 versus the control groups. OA, oleic acid.

In this study, A549 cells and H1299 cells were stimulated with different concentrations of OA for 12 h, and CCK‐8 test was carried out to test the effect of OA on cell proliferation. The results showed that the antiproliferative effect of OA on A549 cells and H1299 cells was dose dependent (Figure 1A,B). Based on the IC50 values of A549 cells and H1299 cells, we chose a concentration of 100 μM for OA, which is also consistent with the concentration used in the study by Jiang [16]. Then we used the same concentration of OA (100 μM) to treat A549 cells and H1299 cells for 6, 12, 24, and 48 h, and the CCK‐8 test showed that the 12‐h cell viability rate was the highest (Figure 1C,D). Therefore, we did a scratch test, and the results showed that compared with that in the control group, the percentage of blank in the OA group was higher (Figure 1E–G). Finally, the MTT experiment showed that the cell viability rate of the OA group was lower than that of the control group (Figure 1H,I). In summary, OA inhibits the proliferation and migration of A549 cells and H1299 cells.

### 3.2 OA Treatment Significantly Inhibits the Expression of SDC4 in A549 Cells and H1299 Cells

**FIGURE 2:**
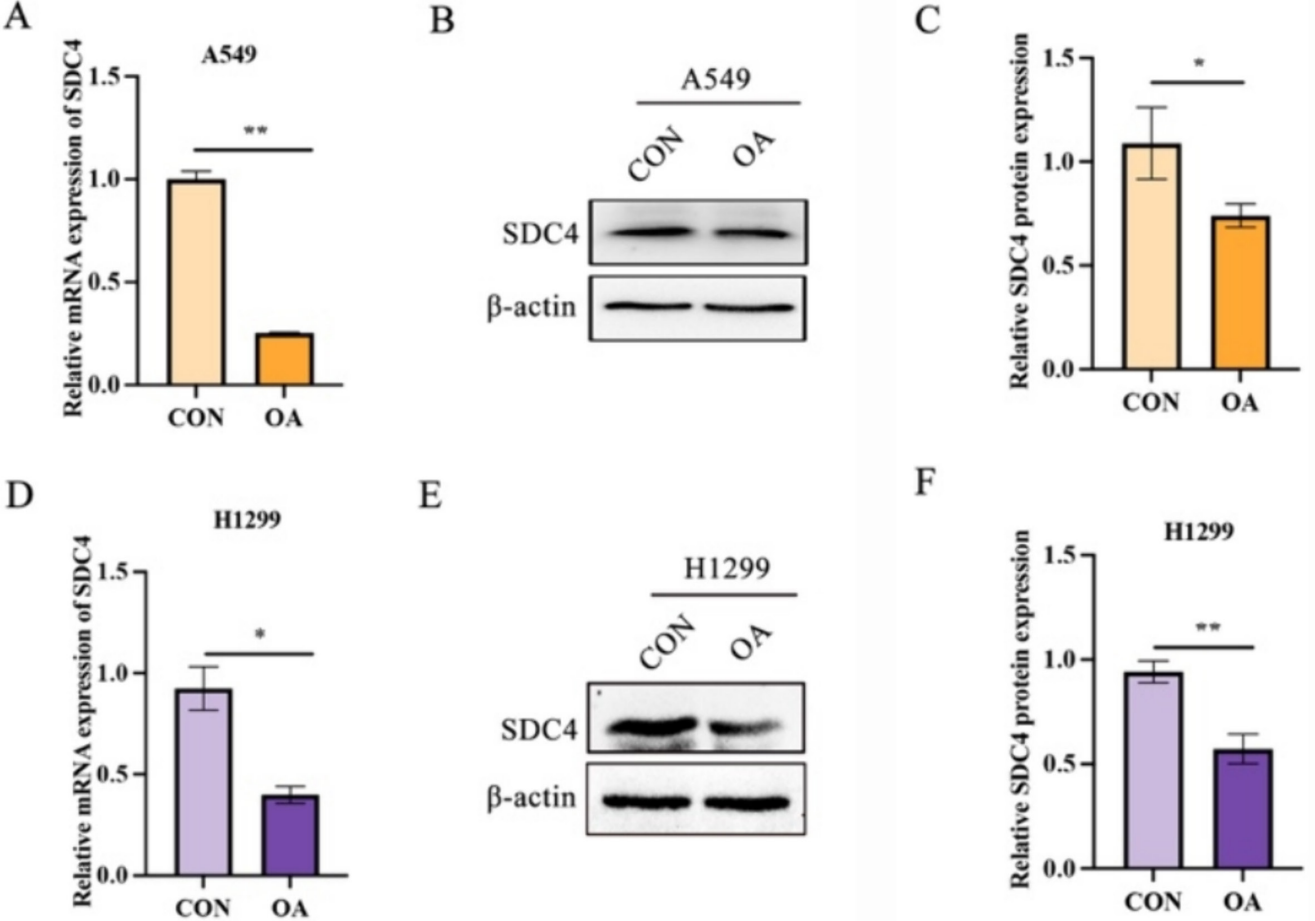
OA treatment significantly inhibits the expression of SDC4 in A549 cells and H1299 cells. (A) Compared with the control group, the expression of SDC4 in A549 cells stimulated by OA decreased at the mRNA level. (B) The expression of SDC4 protein levels in A549 cells stimulated by OA and (C) statistical diagram. (D) Compared with the control group, the expression of SDC4 in H1299 cells stimulated by OA decreased at the mRNA level. (E) The expression of SDC4 protein levels in H1299 cells stimulated by OA and (F) statistical diagram. *n* = 3. *p < 0.05, **p < 0.01 versus the control groups. OA, oleic acid.

The target prediction of molecular docking ligands and structures has been studied. OA's compound corresponds to SDC4, a potential target for OA against NSCLC. Compared with the control group, the expression of SDC4 in A549 cells and H1299 cells was reduced at both mRNA and protein levels under OA stimulation (Figure 2A–F). Therefore, OA treatment significantly inhibited SDC4 expression in A549 cells and H12299 cells.

### 3.3 OA Promotes ROS Release in A549 Cells and H1299 Cells

**FIGURE 3:**
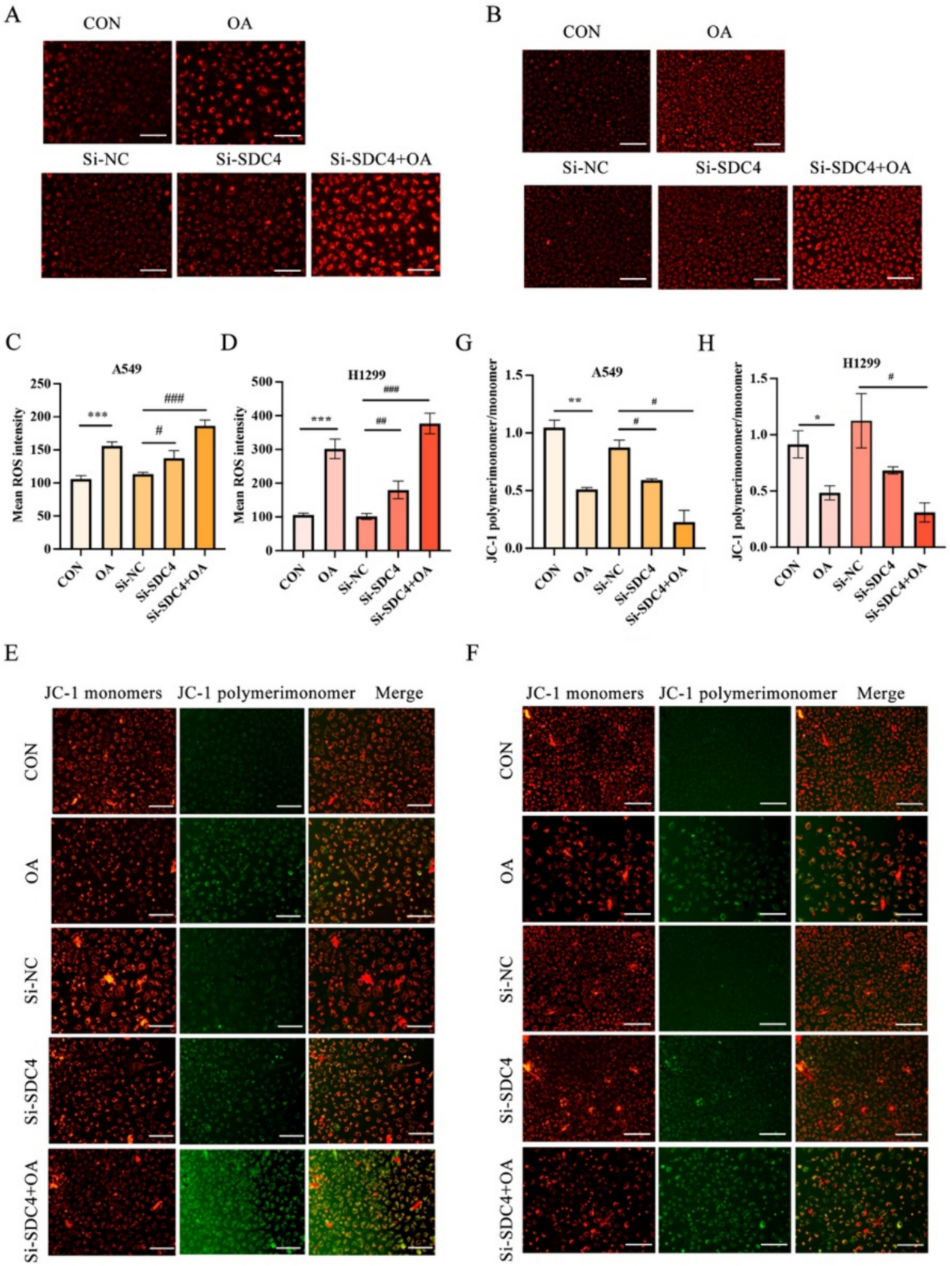
OA promotes ferroptosis of A549 cells and H1299 cells. (A, B) Measurement of ROS in CON, OA, Si‐NC, Si‐SDC4, Si‐SDC4+OA group and (C, D) statistical diagram. (E, F) JC‐1 image detected using confocal laser scanning microscopy (CLSM) and (G, H) statistical diagram. *n* = 3. *p < 0.05, **p < 0.01, ***p < 0.005 versus the control groups. ^#^ *p* < 0.05, ^##^ *p* < 0.01, and ^###^ *p* < 0.001 versus the Si‐NC groups. OA, oleic acid.

We examined ROS in A549 cells and H1299 cells to investigate whether OA induced ferroptosis in A549 cells and H1299 cells, which is the critical event in ferroptosis. The result displayed that OA and si‐SDC4 treatment significantly enhanced the ROS in A549 cells and H1299 cells, while the cotreatment of si‐SDC4 and OA made the ROS expression more obvious (Figure 3A–D). At the same time, the JC‐I probe was used to detect the changes in cell membrane potential to evaluate mitochondrial function, and it was found that the mitochondrial membrane potential decreased after OA and si‐SDC4 stimulation, whereas the membrane potential decreased more significantly when OA and si‐SDC4 were used at the same time (Figure 3E–H). Together, these results reveal that OA could induce ferroptosis in A549 cells and H1299 cells, and this process is closely related to the SDC4 gene.

### 3.4 OA Promotes Ferroptosis Through GPX4/ACSL4

**FIGURE 4:**
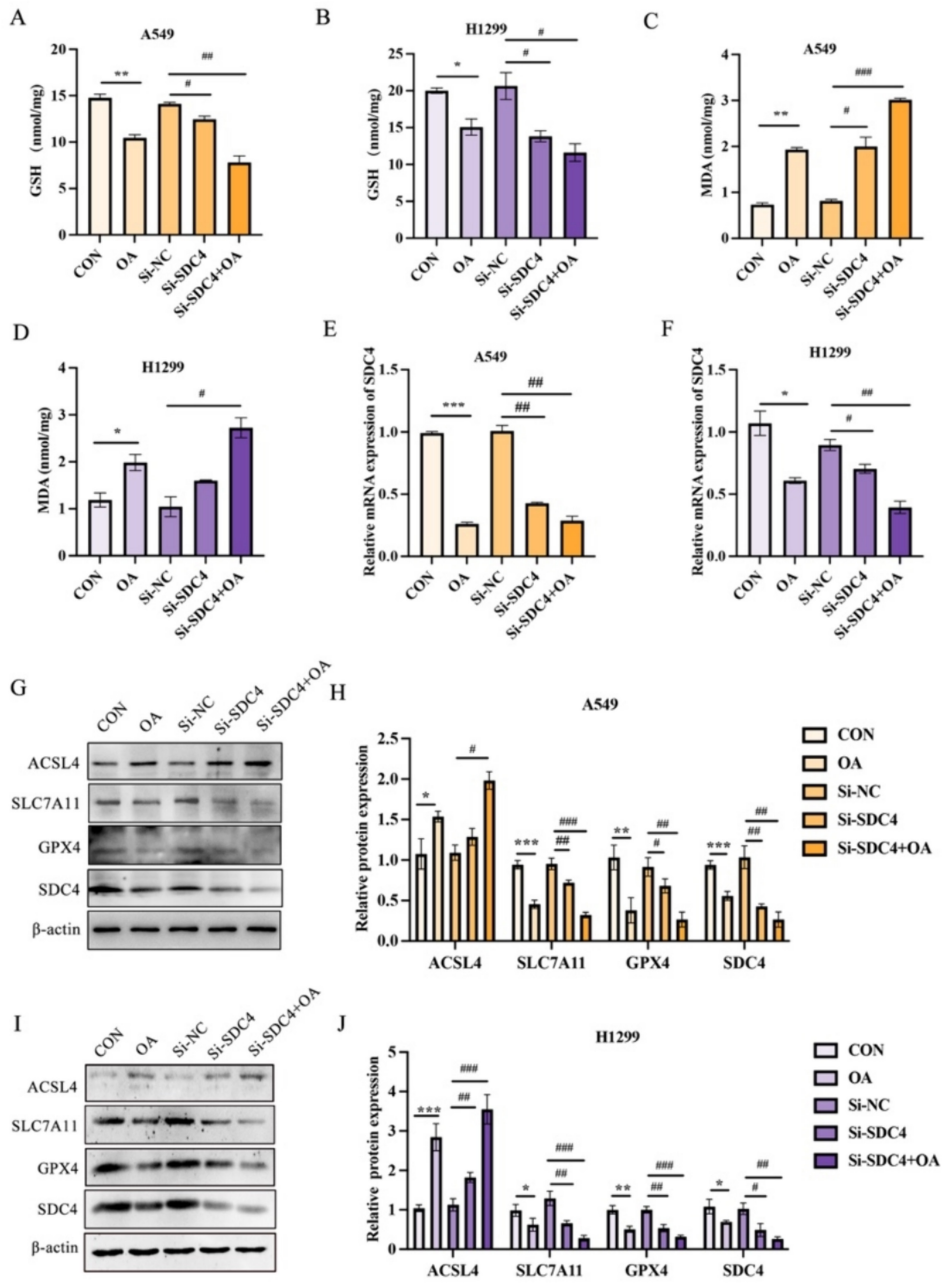
OA promotes ferroptosis through GPX4/ACSL4. (A, B) The expression of GSH in A549 cells and H1299 cells. (C, D) The expression of MDA in A549 cells and H1299 cells. (E, F) The expression of SDC4 at the mRNA level in A549 cells and H1299 cells. (G–J) The expression of ACSL4, SLC7A11, GPX4, and SDC4 at the protein level in A549 cells and H1299 cells and statistical map. *n* = 3. *p < 0.05, **p < 0.01, and ***p < 0.005 versus the control groups. ^#^ *p* < 0.05, ^##^ *p* < 0.01, and ^###^ *p* < 0.001 versus the Si‐NC groups. OA, oleic acid; GSH, glutathione; MDA, malondialdehyde; ACSL4, acyl‐CoA synthetase long‐chain family member 4; SLC7A11, solute carrier family 7 member 11; GPX4, glutathione peroxidase 4.

In this study, we investigated the role of the GPX4/ACSL4 pathway in OA‐induced ferroptosis. Our results showed that OA treatment significantly reduced GSH levels (Figure 4A,B), increased lipid peroxidation levels (Figure 4C,D), and significantly reduced SDC4 mRNA expression (Figure 4E,F). Moreover, we found that OA upregulated ACSL4 expression, a key enzyme involved in lipid metabolism, and reduced the expression of SLC7A11, GPX4, and SDC4 (Figure 4G–J). Furthermore, the combination treatment also results in a significant increase in lipid peroxidation levels compared with the control and single‐treatment groups. This indicated that OA promotes SDC4‐mediated ferroptosis in A549 cells (Figure 4A–J). Collectively, our results demonstrate that OA promotes ferroptosis through the GPX4/ACSL4 pathway, highlighting the potential therapeutic significance of targeting this pathway in ferroptosis‐related diseases.

## 4 Discussion

This study aims to investigate the inhibitory effect of OA on SDC4 and its role in promoting ferroptosis in lung cancer cells. Targeting ferroptosis may be a prospective strategy to combat lung cancer, especially in chemotherapy‐resistant lung cancer patients. Currently, most of the research on inducing ferroptosis has focused on the western medicine pathway; unfortunately, most ferroptosis inducers have been found to be unsuitable for clinical use due to poor water solubility or toxicity [20]. The study of the multitarget properties of the TCM regulating ferroptosis has attracted more attention, which may be the focus of future research. BJO is a common TCM preparation with a variety of pharmacological activities that can be used to treat human immunodeficiency virus (HIV), malaria, tuberculosis, and tumors [21, 22, 23]. Some components of Javanica oil emulsion injection (JOEI) have a special affinity for tumor cell membranes and have potent antitumor activity [24], and JOEI is an effective option for the treatment of patients with NSCLC, which can enhance the clinical efficacy, improve quality of life, and reduce the incidence of platinum‐containing chemotherapy side effects [25, 26, 27]. As the active ingredient of BJO, OA can be widely used in the treatment of various cancers, such as gastric cancer, lung cancer, and liver cancer, with a favorable safety profile. Therefore, a further study of the antitumor mechanism of OA, especially in lung cancer, is important to promote the modernization of OA formulations and the combination therapy in clinical practice.

The SDC4 gene is an important gene that regulates cell adhesion and signaling. It is involved in a variety of pathophysiological processes such as embryonic development, cell migration, inflammatory response, and tumor metastasis [28, 29, 30]. Further research on the functional mechanism of the SDC4 gene will help to deeply understand the regulatory mechanisms of cell adhesion and signaling and provide new targets for disease treatment. Network pharmacology found that NSCLC tissues exhibited a high expression of SDC4, and further study of the interaction between the compounds and the target confirmed that the four compounds of JOEI (linoleic acid, OA, palmitic acid, and stearic acid) showed relatively high potential for binding to the active sites of five targets (FABP4, ABCB1, LDLR, PTGS2, and SDC4), which further verified that the mechanism of JOEI against NSCLC involves multiple targets and signaling pathways [25]. Preliminary results suggest that OA treatment decreases SDC4 expression and enhances lipid peroxidation levels, indicating its potential as a therapeutic method for lung cancer treatment. Moreover, the combination of OA and si‐SDC4 leads to a further decrease in SDC4 expression compared with either treatment alone. This suggests that OA has an inhibitory effect on SDC4.

Ferroptosis has been found to be associated with various diseases, such as cancer, ischemic organ damage, and degenerative diseases. Many ferroptosis inhibitors can be used as indicators of poor prognosis and promote tumor cell growth in lung adenocarcinoma (LUAD) [31, 32, 33]. Ferroptosis occurs in cells, characterized by iron accumulation, ROS production, lipid peroxidation, and decreased GPX4 expression in the antioxidant system (GSH system) [2]. Excess Fe^2+^ reacts with hydrogen peroxide to produce free hydroxide (OH^−^—) and hydroxyl (OH·) radicals, which promote peroxidation of membrane lipids, resulting in ferroptosis [33]. Ferroptosis can also be driven by iron‐dependent phospholipid peroxidation [34]. The imbalance between ROS production and degradation is also one of the key factors [35]. Our results showed that treatment with OA significantly increased lipid ROS levels and lipid peroxidation. Current research results have confirmed the promoting role of TCA (tricarboxylic acid cycle) and ETC (electron transport chain) in ferroptosis, and this phenomenon may be related to changes in mitochondrial membrane potential. In addition, ferroptosis has been found to have a dual impact on cancer immunoregulation as it can not only induce ferroptosis in cancer cells to mediate anticancer immunity but also promote immune escape of neighboring cancer cells and cancer growth, and the communication among cells in the tumor microenvironment (TME) and the tumor cells is also closely related to ferroptosis, which determines the efficacy of ferroptosis induction in vivo together [21, 36, 37, 38]. These are also issues that should be considered in future medical applications.

At present, the antioxidant pathways are the main mechanism regulating ferroptosis; cysteine transporter solute carrier family 7 member 11 (SLC7A11) and GPX4 are its core targets [2]; the SLC7A11‐GSH‐GPX4 pathway is the most and earliest discovered antioxidant system [39]. Targeting GPX4 or SLC7A11 has been shown to reduce tumor growth in different NSCLC model systems [40, 41]. SLC7A11 is overexpressed in a variety of human cancers and induces GSH synthesis by promoting Cys uptake and Glu release, thereby promoting tumor survival. As a key regulator of ferroptosis, the inhibition of SLC7A11 translation can induce ferroptosis, and when the SLC7A11 is upregulated, ferroptosis is inhibited, which is not conducive to lung cancer treatment [2, 42]. Ferroptosis inducers can act directly or indirectly on regulating GSH peroxidases (GPXs) through different pathways, resulting in decreased cellular antioxidant capacity, ROS accumulation, and ultimately cellular oxidative death. GPX4, as a key enzyme in the GSH system, can regulate ferroptosis through multiple pathways. Targeting GPX4 with high expression in LUAD tissues can regulate ferroptosis, thereby affecting the occurrence and progression of lung cancer [43, 44]. Arachidonic acid lipoxygenase 15 (ALOX15), acyl‐CoA synthetase long‐chain family member 4 (ACSL4), and lysophosphatidylcholine acyltransferase 3 (LPCAT3) are key enzymes for lipid metabolism and ferroptosis. Among them, ACSL4 catalyzes the reaction between arachidonic acid (AA)/adrenic acid (AdA) and acyl‐coenzyme A (CoA), and elevated expression levels of ACSL4 were found in patients with highly malignant LUAD, confirming the association of lipid metabolism with ferroptosis in LUAD [45]. Further analysis of GPX4, ACSL4, SLC7A11, and ROS levels confirmed that OA promoted ferroptosis in A549 cells and H1299 cells. Therefore, promoting ferroptosis in lung cancer cells has potential benefits for the treatment of lung cancer.

Although our study confirms the role of OA in inhibiting tumor cells, there are still some limitations. First, this study mainly focused on A549 cells and H1299 cells cultured in vitro, but they cannot completely replace the condition in the lungs, and further studies using animal models and human tissues are necessary to validate the results of this study. Second, our study mainly investigates the role of OA, but the exact molecular mechanisms behind this process remain to be fully elucidated, potentially revealing new therapeutic targets for lung cancer.

In conclusion, our study reveals the role of OA in inhibiting SDC4 expression and inducing ferroptosis in lung cancer cells. Although the current research still has certain limitations, it has the potential to find new therapeutic targets for lung cancer and ultimately improve the prognosis of patients.

## Author Contributions

Conceptualization: Jingfei Dong, Dehui Li, and Yapei Xu. Data curation: Fei Qi. Formal analysis: Yapei Xu and Huiqing Qie. Funding acquisition: Jingfei Dong. Investigation: Shibu Du and Li Li. Methodology: Jingfei Dong and Yan Zhang. Validation: Yapei Xu. Visualization: Fei Qi and Huiqing Qie. Writing – original draft: Jingfei Dong and Kaiyue Xu. Writing – review and editing: Jingfei Dong, Dehui Li, and Yapei Xu.

## Ethics Statement

The authors have nothing to report.

## Conflicts of Interest

The authors declare no conflicts of interest.

## Data Availability

The datasets generated during and/or analyzed during the current study are available from the corresponding author on reasonable request.
